# Prevalence of monarch (*Danaus plexippus*) and queen (*Danaus gilippus*) butterflies in West Texas during the fall of 2018

**DOI:** 10.1186/s12898-020-00301-x

**Published:** 2020-06-12

**Authors:** Matthew Z. Brym, Cassandra Henry, Shannon P. Lukashow-Moore, Brett J. Henry, Natasja van Gestel, Ronald J. Kendall

**Affiliations:** 1 The Wildlife Toxicology Laboratory, Texas Tech University, Box 43290, Lubbock, TX 79409-3290 USA; 2 The Department of Biological Sciences, Texas Tech University, Lubbock, TX USA

**Keywords:** Monarch butterfly, *Danaus plexipuus*, West Texas, Migration, Reproduction, Egg correction

## Abstract

**Background:**

The monarch butterfly (*Danaus plexippus*) is a conspicuous insect that has experienced a drastic population decline over the past two decades. While there are several factors contributing to dwindling monarch populations, habitat loss is considered the most significant threat to monarchs. In the United States, loss of milkweed, particularly in the Midwest, has greatly reduced the available breeding habitat of monarchs. This has led to extensive efforts to conserve and restore milkweed resources throughout the Midwest. Recently, these research and conservation efforts have been expanded to include other important areas along the monarch’s migratory path.

**Results:**

During the fall of 2018, we conducted surveys of monarch eggs and larvae through West Texas. We documented monarch and queen butterfly (*Danaus gilippus*) reproduction throughout the region and used the proportion of monarch and queen larva to estimate the number of monarch eggs. Peak egg densities for monarchs were as high as 0.78 per milkweed ramet after correction for the presence of queens. Despite our observations encompassing only a limited sample across one season, the peak monarch egg densities we observed exceeded published reports from when monarch populations were higher.

**Conclusions:**

To our knowledge, this is the first study to correct for the presence of queens when calculating the density of monarch eggs. This research also provides insight into monarch utilization of less well-known regions, such as West Texas, and highlights the need to expand the scope of monarch monitoring and conservation initiatives. While the importance of monarch research and conservation in the Midwest is unquestionable, more comprehensive efforts may identify new priorities in monarch conservation and lead to a more robust and effective overall strategy, particularly given the dynamic and rapidly changing global environment.

## Background

Monarch butterflies (*Danaus plexippus*) are perhaps the most widely known and recognizable of all insects. These butterflies are a classic example of plant–insect interactions, mimicry, and aposematic coloration [1]. Monarchs are best known, however, for the bi-annual migration of the eastern population between overwintering grounds in central Mexico and summer breeding areas that span from northern Mexico to southern Canada [7, 54]. Monarchs are also well known in the western United States (US), as this area harbors a distinct population that exhibits a similar, albeit less extensive, migration within the region [16]. Unfortunately, the monarch migration is imperiled, both east and west of the Rocky Mountains, due to steep declines in monarch abundance over the past several decades [9, 48].

Since the 1990s, the eastern population of monarchs is estimated to have decreased by ~ 80% [49], while its western counterpart has declined by over 99% since the 1980s [36]. The threats to monarchs are varied and range from extreme weather events [12] and parasites [2, 6] to predation by invasive pests [13] and numerous insect taxa [23]. While many of these factors present substantial threats to monarchs, habitat loss may be the most damaging to overall monarch numbers given the restricted distribution of their overwintering habitat and specialized larval diet [29]. The loss of breeding habitat, in particular, is well supported as a primary cause of monarch declines [52].

In the US, changing agricultural practices and increased herbicide use have led to widespread losses of milkweeds (*Asclepias* spp.), which are an essential food source for monarch larva [39]. Milkweeds in the Midwestern US are considered especially important, as this area has been documented as the primary repopulation zone for monarchs [55]. Because of the monarchs reliance on milkweed, safeguarding and restoring these plants is a top priority for monarch conservation, and it is estimated that ~ 1.6 billion milkweeds must be added to the Midwest in order to reach conservation goals set by the Pollinator Health Task Force [40]. More recently, researchers have also stressed the necessity to expand monarch conservation initiatives beyond the Midwest, as other regions, like the southern US, have been identified as key natal areas for butterflies that go on to colonize summer breeding grounds [18].

Considering the wide spatiotemporal distribution of monarchs, broadening conservation efforts may allow for greater protection of important habitat, offer more area for restoration initiatives, and increase resilience to localized calamities and stochastic variability. A broader focus could also help to distribute the costs associated with monarch conservation across a wider base, allowing for the mobilization of more resources towards milkweed propagation and restoration, habitat conservation, monarch monitoring, etc. Indeed, while the southern and north central portions of the monarchs breeding range are regarded as a priority, there is also agreement that an investment in conservation efforts across the entirety of the monarch’s migratory distribution would likely yield the most effective strategy to mitigate monarch declines [19, 20, 34].

However, despite the potential benefits of a comprehensive strategy for monarch conservation, there are also obstacles which impede the implementation of such an approach. For example, there are over 130 species of milkweeds growing across North America [17, 58], and these may require different cultivation techniques and growing conditions [27]. Milkweed may also be unavailable commercially, making large scale conservation and restoration initiatives difficult in areas where local plant ecotypes are scarce [5]. Determining what species of milkweeds to select and how to distribute them also presents a challenge, as studies show that monarch utilization is affected by site and landscape characteristics [22, 38, 62], ovipositing females prefer some milkweed species over others [3, 25, 42, 43], and larval success varies with milkweed species as well [25, 41, 60].

Ultimately, a significant barrier to more widespread monarch conservation is an incomplete understanding of the factors affecting monarch success and habitat utilization. Addressing this requires comprehensive monitoring, and while much effort has been focused on the Midwest [53], research into other areas along the monarch’s migratory route is more limited. The limitations within the knowledge base are present even in areas considered to be highly significant to monarch conservation, like Texas. Although Texas has several monarch monitoring programs such as Texas Monarch Watch [44], growing coverage due to citizen science programs [24], and surveys by Calvert and Wagner [14], there are still gaps in our understanding of how monarchs utilize resources within the state. This is especially true for the western portion of the state, which is sparsely populated and oftentimes overlooked in comparison to the rest of Texas [10]. However, recent surges in monarch abundance through West Texas may offer insight into the significance of this region that warrants further investigation. In this study, we examine surveys of monarch egg and larval abundance from West Texas during the fall of 2018. If monarch abundance in West Texas is comparable to that of more widely recognized and monitored regions, it may be worthwhile to look more closely at the significance of this area in terms of monarch conservation.

## Results

### Proportion of eggs based on larva

The proportion of monarch larva observed was consistently higher than that of queen butterfly (*Danaus gilippus*) larva across the majority of our study sites and survey sessions (Table 1). There were only 6 of the 48 surveys where queen larva exceeded that of monarchs, and these only occurred on 2 of the sites. During September 30th, an equal number of monarch and queen larva were observed at Stonewall 3, and this was also the case at both Fisher 1 and Stonewall 2 on October 22nd. The number of monarch and queen eggs based on these proportions and confidence intervals is summarized in Table 2. It is important to note that estimating the number of monarch eggs by multiplying total eggs observed by the proportion of monarch to queen larva does not consider factors such as differing egg and larval survival rates between the two butterfly species. However, because we found no published comparisons of survival rates between immature queen and monarch butterflies, and rearing eggs for positive identification was beyond the scope of this study, we were unable to more precisely estimate the number of monarch eggs observed. Nevertheless, the close relationship and similar life histories of the two butterflies suggest that our estimates of monarch eggs were generally representative. This is supported by a study that found the immature survival rates of monarchs and another congeneric species, the African queen (*Danaus chrysippus*), to be similar [59].

**Table 1:** Summary of monarch and queen proportions

Date	Species	Fisher 1		Fisher 2		Fisher 3		Stonewall 1		Stonewall 2		Stonewall 3	
		P	CI	P	CI	P	CI	P	CI	P	CI	P	CI
9/14/18	M	0.86	0.74–0.97	0.75	0.59–0.91	0.90	0.71–1.09	1.00	NA	0.64	0.35–0.92	1.00	NA
	Q	0.14	0.03–0.26	0.25	0.09–0.41	0.10	−0.09–0.29	0	NA	0.36	0.08–0.65	0	NA
9/24/18	M	0.62	0.44–0.80	0.95	0.89–1.02	0.81	0.66–0.96	1.00	NA	0.39	0.19–0.59	0.83	0.54
	Q	0.38	0.20–0.56	0.05	–0.02–0.11	0.19	0.04–0.34	0	NA	0.61	0.41–-0.81	0.17	−0.13–0.46
9/30/18	M	1.00	NA	0.81	0.64–0.98	0.88	0.71–1.04	1.00	NA	0.36	0.08–0.65	0.50	–0.19–1.19
	Q	0	NA	0.19	0.02-0.36	0.13	−0.04–0.29	0	NA	0.64	0.35–0.92	0.50	−0.19 –1.19
10/5/18	M	0.90	0.71–1.09	0.73	0.51–0.96	0.65	0.42–0.87	1.00	NA	0.44	0.19–0.68	0	NA
	Q	0.10	−0.09–0.29	0.27	0.04–0.49	0.35	0.13–0.58	0	NA	0.56	0.32–0.81	1.00	NA
10/12/18	M	0.86	0.60–1.12	0.77	0.54–1.00	0.60	0.17–1.03	NLO	NA	0.71	0.38–1.05	0.17	−0.13 –0.46
	Q	0.14	−0.12–0.40	0.23	0–0.46	0.40	−0.03 –0.83	NLO	NA	0.29	−0.05–0.62	0.83	0.54–1.13
10/22/18	M	1.00	NA	0.67	0.13–1.20	0.50	−0.19–1.19	1.00	NA	0.50	0.15–0.85	0	NA
	Q	0	NA	0.33	−0.20–0.87	0.50	−0.19–1.19	0	NA	0.50	0.15–0.85	1.00	NA
10/29/18	M	1.00	NA	1.00	NA	1.00	NA	NLO	NA	0.60	0.17–1.03	NLO	NA
	Q	0	NA	0	NA	0	NA	NLO	NA	0.40	−0.03–0.83	NLO	NA
11/9/18	M	NLO	NA	NLO	NA	NLO	NA	NLO	NA	1.00	NA	NLO	NA
	Q	NLO	NA	NLO	NA	NLO	NA	NLO	NA	0	NA	NLO	NA

Proportions (P) of monarch (M) and queen (Q) butterfly larvae with 95% confidence intervals (CI) by date and site. Confidence intervals were not available (NA) when there were either no larvae observed (NLO) or there was only one species of larvae observed

**Table 2:** Summary of estimated monarch and queen eggs

Date	Fisher 1			Fisher 2			Fisher 3			Stonewall 1			Stonewall 2			Stonewall 3		
	M	Q	±	M	Q	±	M	Q	±	M	Q	±	M	Q	±	M	Q	±
9/14/18	25	15	7	92	5	6	54	13	10	20	0	NA	2	2	1	6	1	2
9/24/18	7	0	NA	9	2	2	9	1	2	7	0	NA	10	18	8	1	1	1
9/30/18	33	4	7	12	4	4	8	4	3	8	0	NA	5	6	3	0	5	NA
10/5/18	5	1	2	1	0	NA	2	2	2	0	0	NA	3	1	1	1	3	1
10/12/18	0	0	NA	0	0	NA	0	0	NA	0	0	NA	0	0	NA	0	0	NA
10/22/18	2	0	NA	0	0	NA	0	0	NA	0	0	NA	11	8	8	0	0	NA
10/29/18	0	0	NA	0	0	NA	0	0	NA	0	0	NA	6	0	NA	0	0	NA

Estimated number of monarch (M) and queen (Q) eggs based on the subsequent weeks proportion of larvae with ± representing the calculated confidence interval for the proportions by date and site. Confidence intervals were not available for all proportions and this is represented by NA

### Comparison of abundance

**Fig. 1:**
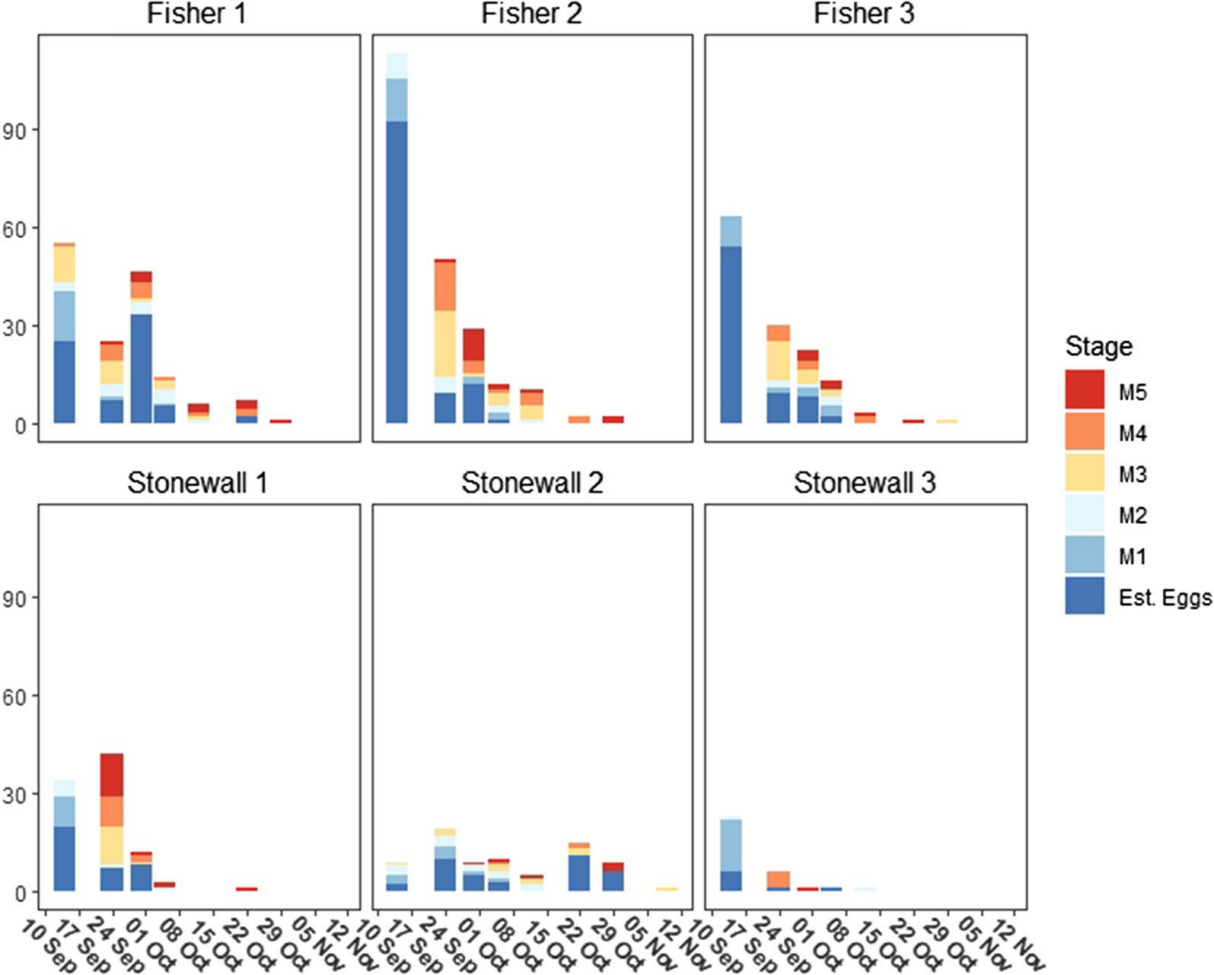
Estimated monarch eggs and larva by location and date. Visual representation of estimated monarch eggs and observed larva for each study location throughout the survey period. Designations for the first through fifth instar larva have been labeled M1–M5, respectively

**Fig. 2:**
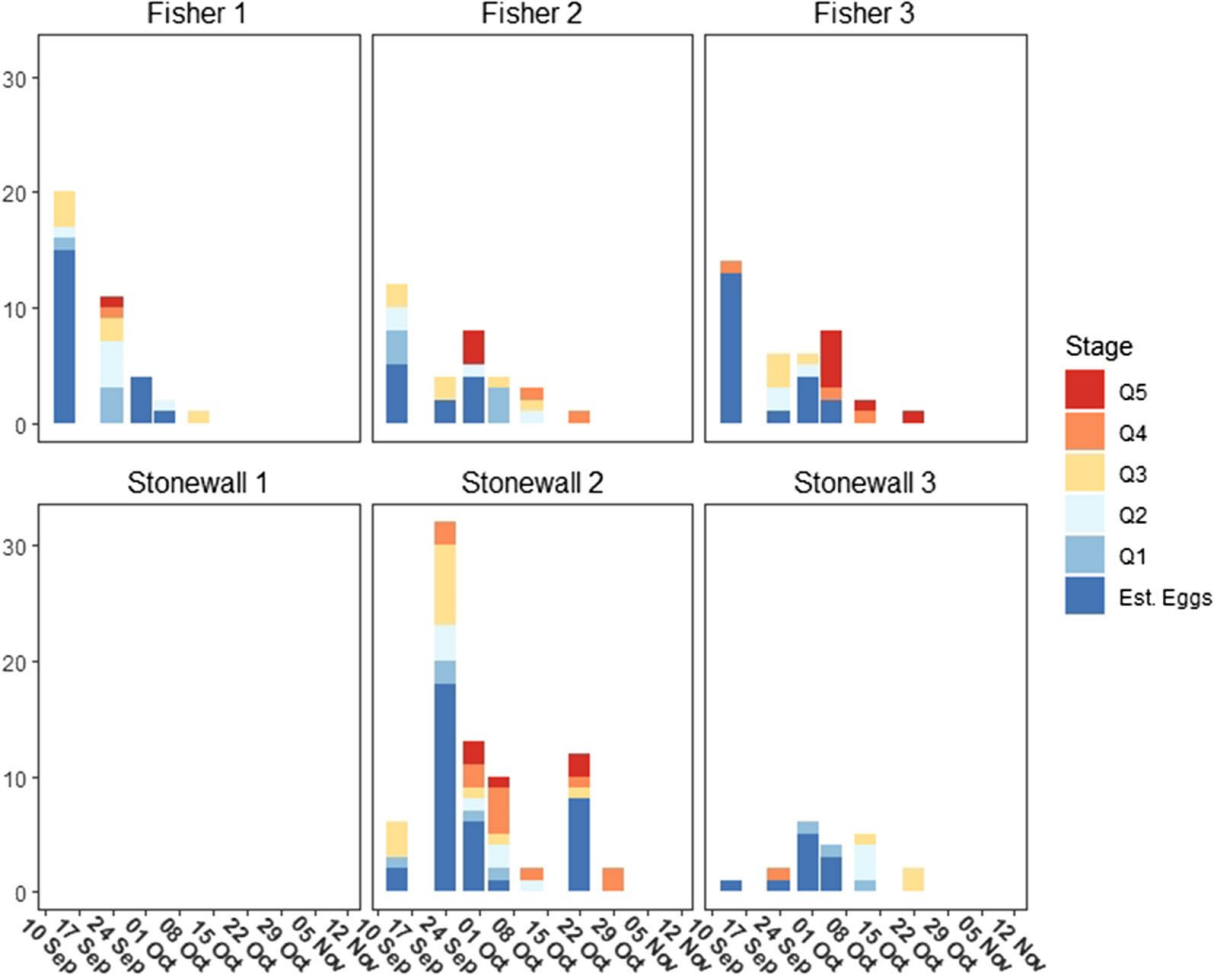
Estimated queen eggs and larva by location and date. Visual representation of estimated queen eggs and observed larva for each study location throughout the survey period. Designations for the first through fifth instar larva have been labeled Q1–Q5, respectively

**Fig. 3:**
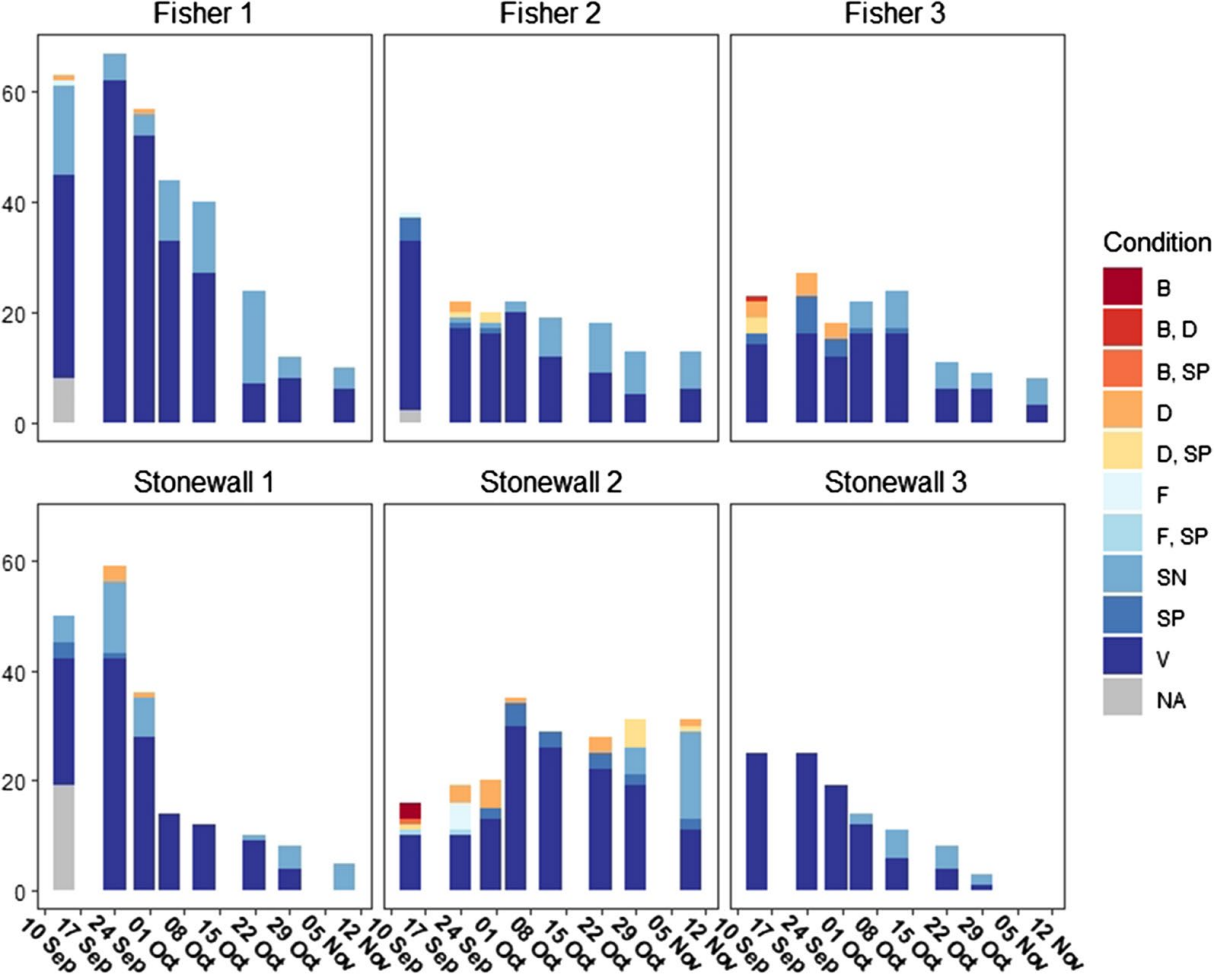
Milkweed condition by location and date. Stacked bar graphs representing the condition (*B* Budding, *D* Dehiscent, *F* Flowering, *SP* with Seedpod, *SN* Senescing, *V* Vegetative) of milkweed throughout the survey period by location

Across the two counties, monarch egg and larva abundance generally followed a downward trend, with a few exceptions that can be visualized in Fig. 1. Monarch eggs and larva were also more abundant in Fisher County overall. In contrast, queen eggs and larva were most abundant at Stonewall 2 but appeared to be more evenly distributed throughout the Fisher County sites (Fig. 2). As the sampling period progressed, there were fewer plants sampled with a higher proportion of senescing plants (Fig. 3).

**Fig. 4:**
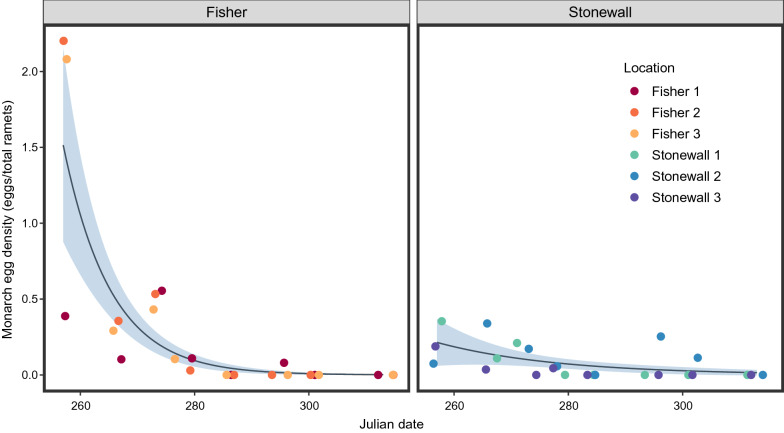
Monarch egg density as a function of Julian date. Temporal trends for monarch egg densities. The trends were significant for both Fisher and Stonewall County based on the best fitting GAMM model, the blue region represents the 95% confidence bands of the fitted line. The “geom_jitter” function was used in R to account for overplotting and allow for easier visualization of data points

Peak abundance and maximum average density of monarch eggs occurred during the first survey on September 14th when a total of 235 *Danaus* eggs were counted across 6 sites and ~ 240 milkweed ramets (Table 3). After correcting for the number of queen eggs, we estimated ~ 187 monarch eggs were observed during this session resulting in an overall density of ~ 0.78 monarch eggs per milkweed ramet. Over the course of the study period, 1307 milkweed ramets were surveyed for monarchs across 6 sites and 8 monitoring sessions. The number of milkweed ramets examined averaged 163 ± 57 per session and ranged from a maximum of 245 on September 24th to a minimum of 83 on November 9th. The best supported model of the candidate models for estimated monarch egg density included only Julian date (Table 4), with egg density decreasing over time across both Fisher (p < 0.0001) and Stonewall (p = 0.0044) counties (Fig. 4). This model was chosen because it had the lowest AIC_c_ and a *w*_i_ of 0.768. Because the second-best model had a Δ_*i*_ of 3.01, we did not use model averaging.

**Table 3:** Summary of monarch egg and larva surveys

Date	Milkweed ramets surveyed	Total estimated monarch eggs	Estimated monarch eggs per ramet
9/14/2018	240	187	0.78
9/24/2018	245	51	0.21
9/30/2018	189	59	0.31
10/5/2018	176	13	0.07
10/12/2018	151	0	0
10/22/2018	122	16	0.13
10/29/2018	101	6	0.06
11/9/2018	83	0	0
Total	1307	332	0.25

Average number of milkweed ramets surveyed and monarchs observed per milkweed ramet over 8 weekly sessions in West Texas during the fall of 2018

**Table 4:** Summary of candidate models

Candidate model	Fixed effects of candidate models	*Df*	AIC_c_	Δ*i*	*W*_i_	*E*_i_
Model 1	Julian day	7	187.56	0	0.768	1
Model 2	Julian day + Plot area	9	190.58	3.01	0.170	5
Model 3	Julian day + Area size	9	194.24	6.68	0.027	28
Model 4	Julian day + Ramet density	9	194.39	6.83	0.025	30
Model 5	Julian day + Ramet density + Plot area	11	197.31	9.75	0.006	131
Model 6	Julian day + Area size + Plot area	11	198.57	11.01	0.003	246
Model 7	Julian day + Ramet density + Area size	11	201.78	14.22	0.001	1222
Model 8	Julian day + Ramet density + Area size + Plot area	13	206.80	19.24	0	15,035
Model 9	Area size	5	220.49	32.92	0	>10^6^
Model 10	Plot area	5	221.03	33.47	0	>10^6^
Model 11	Area size + Plot area	9	225.76	38.20	0	>10^7^
Model 12	Ramet density + Area size + Plot area	7	226.80	39.24	0	>10^7^
Model 13	Ramet density	5	227.07	39.51	0	>10^7^
Model 14	Ramet density + Area size	7	233.97	46.41	0	>10^9^
Model 15	Ramet density + Plot area	7	234.87	47.31	0	>10^9^

The set of candidate models used in the model selection procedure to predict monarch egg density (eggs/milkweed ramet). All candidate models are generalized additive mixed models with site (Fisher 1–3 and Stonewall 1–3) as the random intercept. Our predictor variables were Julian day, plot area, area size, and ramet density (ramets/m^2^). We considered all possible combinations of these predictor variables. Smoothers were applied to Julian day (at the county level), plot size, and ramet density. The results are based on a negative binomial distribution because of overdispersion. Results presented include the degrees of freedom (df), corrected AIC (AIC_c_), the AIC_c_ difference between the best model and all other models (Δ*i*), Akaike weights (*w*_i_), and the evidence ratio (*E*_*i*_*)*. The results are ranked by AIC_c_, from the best to the worst model

## Discussion

With the increased attention and effort given to the protection of monarchs, as well as emphasis towards conservation initiatives across a wider portion of their migratory range [19, 20, 34], it is necessary to enhance our understanding of the phenology and migratory dynamics of this butterfly. This is particularly important given the threat of climate change and its potential to affect the abundance, distributions, and habitats of migratory organisms [46] like the monarch [26]. In this study, we observed substantial utilization of milkweed resources and reproduction of monarchs, with all larval stages documented in West Texas during the fall of 2018. This coincided with a surge of citizen scientist reports of monarchs following a more westerly distribution, with concentrations of butterflies being found as far west as Colorado and New Mexico [24]. Although West Texas has limited monarch monitoring initiatives on account of its sparse population and location at the edge of the migratory corridor [10], our results suggest that further examination of monarch utilization in this area may be warranted.

This assessment of monarch activity in West Texas also provides a preliminary account of how monarchs utilize milkweed resources within the region during the fall. We noted a high degree of variability in monarch utilization between sites which was consistent with that of other studies [22, 38, 51]. We also noted a potential difference in the utilization of milkweed species between queen and monarch butterflies, as queens were disproportionately distributed at Stonewall 2. The disproportion of queens may be due to this site being the only one that contained zizotes milkweed (*Asclepias oenotheroides*). Although one site with zizotes is insufficient to evaluate the preference of milkweed species by monarchs and queens in Texas, it does highlight the need to further study this dynamic. Additionally, the differences between study locations during different sampling periods emphasize the need to account for the proportion of queen eggs when calculating monarch egg densities, which are a standard for assessing the utilization of milkweed resources in an area. If queen eggs are not accounted for, there is the potential for this to significantly affect estimates of monarch utilization in areas, like Texas, where the two species are sympatric.

Because monarchs are influenced by the availably of milkweed and flowering plants along their migratory routes, dynamic weather patterns that shift the distribution of these resources may likewise affect their migration [4, 26, 61]. Models also suggest that the distributions of both monarchs and milkweed are limited by precipitation and temperature, with the distribution of milkweed being a strong predictor of monarch observations [26]. The successive northward expansion of monarchs during the spring is an example of the coadaptation of monarchs and milkweed to avoid increasing temperatures and deteriorating milkweed resources in southern areas [30]. Given the delayed and more gradual onset of winter in the US due to climate change [37], it may also be pertinent to consider the possibility of similar southward movement that precedes the main migration of reproductively inactive adults. This is intriguing, as the peak abundance of monarch eggs we observed on September 14th preceded the height of the monarch migration through our study area, which occurred on October 10th [24]. The following survey on September 24th yielded the highest number of monarch larva counted during the study, suggesting that conditions were favorable for egg hatching and larval development. Overall milkweed quality and the total number of ramets observed was also the highest during this time, potentially due to increased precipitation in West Texas during the early autumn of 2018 [32]. Thus, the monarch breeding we observed may have been a response to plentiful milkweed resources promoted by increased precipitation in West Texas, and further research may provide insight into the importance of such opportunities, as well as the ability of monarchs to find and exploit them.

Increasing regional temperatures [31] may have further contributed to the amount of monarch breeding we observed. Temperature is an important cue governing reproductive diapause in monarchs [21] and higher temperatures could have broken diapause in migrants from further north and/or delayed the onset of diapause in butterflies with more southerly origins. The potential of increased temperature to extend the period of monarch reproductive activity has been highlighted before [21, 26], and this may explain our observations of additional, albeit smaller, peaks in monarch reproduction into October, during which we would expect monarchs to be in diapause.

While assessing the impacts of phenological shifts of host plants and climate change on monarchs was beyond the scope of this study, our observations emphasize the need to further investigate these dynamics, as they may have profound effects on the monarch’s migratory cycle. Such climatic variability could positively impact monarchs as increased abundance of host plants and higher fall temperatures along the southern extent of the monarch’s migratory range may allow for an additional generation, thereby causing this region to serve as a source for monarch populations. Conversely, if monarchs along their southward migration are reproductively active but there is not enough time for their offspring to mature before the onset of winter and adults expend energy essential for overwintering on breeding, the southern portion of the monarch’s migratory range would act as an ecological trap for the butterflies.

Continued monitoring of monarchs in West Texas is therefore necessary to develop our understanding of how monarchs utilize resources within this region, as well as provide greater insight into this particular stage of the monarch’s migration. These efforts may also allow us to better assess the significance of the western extent of the monarch’s migratory corridor compared to other areas. During the peak of monarch activity in our study area, we documented an average of 0.78 monarch eggs per milkweed ramet. This was higher than reported by Stenoien et al. [51] during 14 of their 17 years assessing fall monarch egg densities in the southcentral US, which included sites from central and eastern Texas but lacked any in West Texas. Additionally, the densities reported here were higher than 8 of 14 years of spring densities in the southcentral US, 17 of 18 years of spring densities in the northcentral US, and all 18 years of summer densities in the northcentral US [51]. However, monarch egg densities from the southcentral US, in particular, were subject to a wide degree of variance [51], and because our sample was relatively small and limited to only 1 year, the higher egg densities we observed may have been due to stochastic variability.

The comparison between our data set and Stenoien et al. [51] is further limited as the latter was taken years before our study and encompassed different phases of the monarch’s migratory cycle. An additional caveat of comparing monarch egg densities between regions is the fact that monarch egg density per ramet does not necessarily translate into monarch production. For this, we would also need to consider the total number of milkweed over which this density is distributed. It is therefore imperative to note that the comparison of the abundances between this study and Stenoien et al. [51] should not be taken as evidence of greater monarch production in our area. As such, a larger data set from West Texas that is taken over a greater temporal scale and consistent with monitoring of other regions is necessary in order to achieve a more robust comparison. Nevertheless, it is worth considering the increase we observed because Stenoien et al. [51] evaluated data from as early as 1997 when monarch populations were higher, and they noted that monarch egg densities were declining after 2006. Consequently, we would expect lower egg densities associated with reduced populations, and our findings may have been influenced by factors that warrant future investigation, such as crowding due to reduced milkweed numbers or phenological shifts.

## Conclusion

The wide migratory distribution of the monarch butterfly presents many opportunities to facilitate the conservation of this iconic species. Unfortunately, many areas, like West Texas, may have the potential to benefit monarch conservation that is undermined by a limited knowledge of local milkweed abundance and monarch utilization. While monarch research and protection initiatives are steadily increasing, many of these efforts are still centered on summer breeding areas in the Midwest because the Midwest has among the largest numbers of milkweed, making it a primary source of monarch production [40]. Indeed, we do not argue the significance of the Midwest in terms of monarch conservation. Rather, we emphasize the need to continue expanding conservation efforts outside such prominent regions in pursuit of a more comprehensive approach. This approach would allow the mobilization of resources across a greater base, resulting in more widespread and effective outcomes for monarch conservation, while potentially identifying new priorities in monarch conservation that may arise in our ever-changing world. As such, we hope that this work helps to encourage research and conservation across the entirety of the monarch’s migratory range.

## Methods

### Study area

Monarchs and milkweed were monitored on private ranches in Stonewall County and Fisher County, Texas from September 14th to November 9th, 2018. Both ranches are at the western extent of the monarch migratory corridor and consist of semi-arid rangeland typical of West Texas. The predominant vegetation in this area includes juniper (*Juniperus pinchotti*), honey mesquite (*Prosopis glandulosa*), lotebrush (*Ziziphus obtusifolia*), prickly pear (*Opuntia* spp.), and silver bluestem (*Bothriochloa saccharoides*), with a further description of the region provided by Rollins [47]. West Texas hosts a number of milkweed species, including antelope horn milkweed (*Asclepias asperula*), broad leaf milkweed (*A. latifolia*), and zizotes milkweed (*A. oenotheroides*) [50], which provide breeding habitat for monarchs. Additionally, nectar plants in Texas are considered to be a crucial source of lipids for overwintering monarchs [8], and several species of fall blooming wildflowers, including sunflowers (*Helianthus* spp.), cowpen daisy (*Verbesina encelioides*), and Illinois bundleflower (*Desmanthus illinoensis*), occur in our study area [47].

### Surveys

**Fig. 5:**
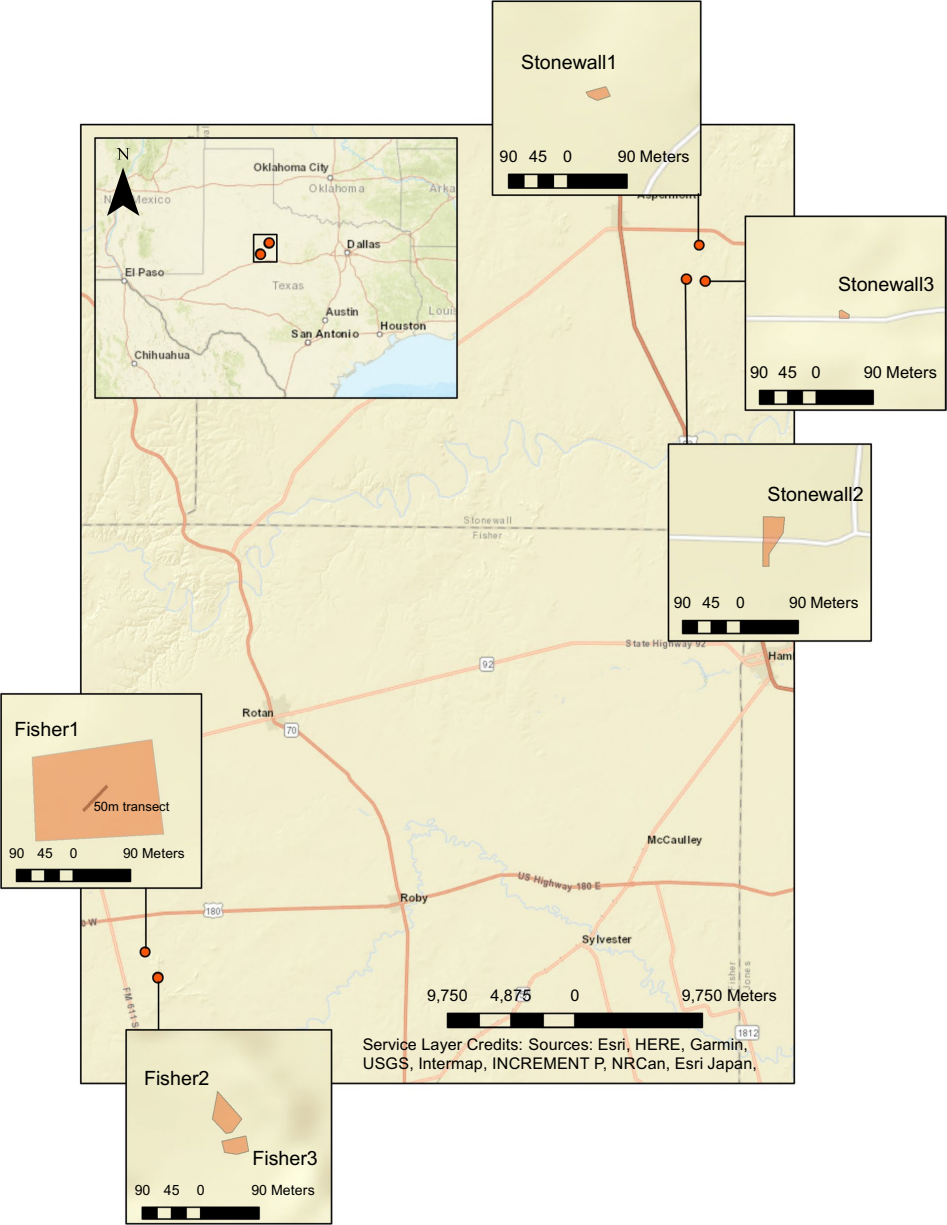
Map of survey locations. Map depicting the location of the survey counties with respect to their location in Texas (top left). The relative sizes and locations of the Stonewall County survey sites are displayed at the top right and Fisher County site locations and relative sizes are bottom left. Milkweed were only surveyed along the 50 m x 4 m transect in Fisher1 due to the immense size of the plot. This figure was created by the authors using ArcMap version 10.8 (https://desktop.arcgis.com/en/arcmap/)

Surveys of monarchs were based on methods utilized by the Monarch Larva Monitoring Project [28]. Monitoring was conducted every 5–10 days at 3 sites per ranch, apart from the final survey which was separated from the previous session by an 11 day interval due to a logistical constraint. The term site(s) will hereafter refer to the 6 survey locations (Fisher 1–3 and Stonewall 1–3). Sites consisted predominantly of indigenous broadleaf milkweed patches and were separated by at least 1 km, except for 2 sites in Fisher County which only had an ~ 30 m separation and 1 site in Stonewall County which also contained another species of milkweed, zizotes (Fig. 5). All milkweed at each site were surveyed and the species of milkweed, condition of plants (budding, dehiscent, flowering, with seedpod, senescing, and/or vegetative), number of ramets (individual stems denoted by a separation of earth between them), number of monarch and queen butterfly eggs, number and stage of monarch larva, and number and stage of queen butterfly larvae were recorded. At one site in Fisher County, there were more plants than could be feasibly surveyed; therefore, a line transect method was used [15] with all milkweed within a 50 m x 4 m plot being surveyed as a representative sample for the local milkweed population (Fig. 5).

It should be noted that for Stonewall 1 on September 14th the number of ramets of some milkweed were not recorded if there were no eggs or larvae present, but the presence of those milkweed were noted. To maintain a larger sample size, the ramets that were not recorded were substituted with the average ramets calculated using the complete records from that date and site. We are confident that this is representative of the ramets considering > 90% of the milkweed at that time and site had only one ramet. Using this substitution, we calculated an overall density of ~ 0.78 monarch eggs per milkweed ramet for September 14th. Alternatively, we excluded the site with incomplete ramet data and this produced an overall density of ~ 0.93 monarch eggs per milkweed ramet. To provide the most representative estimate of monarch egg density for our study area, we chose to use the smaller value achieved by supplementing the data with averages rather than excluding the site. Given our limited sample size, omitting the data from the entire site would have considerably impacted our results and may have inflated the egg density estimates we used for comparison.

### Proportion of eggs based on larva

Because monarchs are sympatric with queen butterflies in our study areas [35] and the eggs of the 2 species appear identical, we corrected for the number of queen eggs during each survey to prevent over representing monarch abundance. This was done by counting both monarch and queen larvae, which can be distinguished from each other by the number of tentacles present [35], and then dividing the number of monarch larvae by the total larvae to calculate the proportion of monarchs and queens. Confidence intervals for the proportion of monarchs and queens were calculated for each site and sampling period. The total number of eggs observed during each survey was then multiplied by the proportion of monarch and queen larvae from the following survey to produce the corrected number of monarch and queen eggs, respectively. For example, the total number of eggs counted on September 14th was multiplied by the proportion of monarch larvae observed on September 24th, to produce the corrected number of monarch eggs for September 14th. Estimating the number of monarch eggs in this manner was done to account for the time it would take the eggs to develop into larvae, as monarch eggs require ~ 45 degree days above a developmental zero of 11.5 °C to hatch [59], which typically takes ~ 4 days under suitable field conditions [33]. The upper and lower confidence intervals of the proportions were also used to give a range of the possible monarch and queen eggs.

### Comparisons of abundance

To illustrate variability between sites and changes in egg and larval distributions over time, stacked bar graphs of all monarch larva stages and estimated monarch eggs for each sampling period and site were generated in RStudio (version 1.2.5033; [45]) using the ggplot2 package [56]. The same was done for queen butterflies. Stacked bar graphs for the condition of all milkweed plants surveyed for each sampling period and site were also generated to illustrate changes in plant abundance and quality over time. Note that on September 14th the total number of milkweed surveyed was available, but plant condition data for Fisher 1 and Stonewall 1 was incomplete. To best represent the data, these data were included in the bar graphs as not applicable (NA).

Then we used generalized additive mixed models (GAMM; mgcv package; [57]) to determine which factors were important in predicting monarch egg density. Site was used as a random intercept because the same sites were checked at each monitoring session over the course of the study, resulting in repeated samples that were not independent. We hypothesized that Julian day, ramet density (ramets/m^2^), plot size, and area size were important predictors of monarch egg density, and included these as predictor variables within the GAMM. We used the additive model approach because of the non-linear behavior between monarch egg density across time. Because of the overdispersion of the non-zero density data, the additive model performed better with a negative binomial distribution than a gaussian distribution. We used smoothers for all the data except for the categorical variable (area size). Because of the distinct temporal pattern between the locales (Fisher versus Stonewall), we applied the smoothers for Julian date at the county level. We compared all possible combinations of the predictor variables, which resulted in 15 GAMMs (Table 4). We then calculated Akaike Information Criterion (AIC), corrected AIC (AIC_c_), Akaike weights (*w*_i_), and evidence ratio (*E*_*i*_) to select the best model [11].

The AIC is calculated from the maximum likelihood estimate of the model and the number of *k* fitted parameters. The equation for AIC is as follows (1):

$$AIC\, = \, - 2In\left( L \right)\, + 2k$$

We then corrected AIC because of the number of observations relative to the number of fitted parameters. Where AIC and *k* are as before (1), and *n* is the sample size. The equation for AIC_c_ is as follows (2):

$$AIC_{c\,} \, = \,AIC\, + \frac{{2k\left( {k\, + \,1} \right)}}{n - k - 1}$$

We calculated Akaike weights for each model (*w*_i_) from the difference in AIC_c_ values between the best model (i.e., with lowest AIC_c_) and all other models in the candidate set (Δ*i*). Where *N* is the total number of candidate models. The *w*_i_ have values ranging between 0 and 1 and can be interpreted as the probability that a given model is the model that predicts the data the best of the candidate models considered. The equation for *w*_i_ is as follows (3):

$$w_{i} \, = \,\frac{{\exp \,\left( { - 0.5\Delta_{i} } \right)}}{{\sum\limits_{n = 1}^{N} {\left( { - 0.5\Delta_{n} } \right)} }}$$

Lastly, the evidence ratio (*E*_i_) is a measure of how much more likely the best model (with weight *w*_best_) is compared to all other models. For example, if the next-best model has *E*_i_ of 2 then the first (best) model is twice as likely to be the best approximating model. The evidence ratio can be computed based on the Akaike weights as follows (4):

$$E_{i} \, = \,\frac{{w_{best} }}{{w_{i} }}$$

In order to provide a broader context of monarch abundance in West Texas, data from all 6 sites were pooled. We calculated the average density of monarch eggs per ramet for each session by dividing the estimated number of monarch eggs by the total number of milkweed ramets surveyed. Maximum average monarch egg density was compared to published data from the northcentral, northeastern, and southcentral US taken from Stenoien et al. [51].

## Data Availability

The datasets used and/or analyzed during the current study are available from the corresponding author on reasonable request.

## Abbreviations

AIC: Akaike information criterion
AIC_c_: Corrected akaike information criterion
*E*_*i*_: Evidence ratio
GAMM: Generalized additive mixed model
NA: Not applicable
US: United States
*w*_i_: Akaike weights
